# Personalized prediction of delayed graft function for recipients of deceased donor kidney transplants with machine learning

**DOI:** 10.1038/s41598-020-75473-z

**Published:** 2020-10-27

**Authors:** Satoru Kawakita, Jennifer L. Beaumont, Vadim Jucaud, Matthew J. Everly

**Affiliations:** Terasaki Research Institute, Los Angeles, CA USA

**Keywords:** Prognostic markers, Outcomes research, Translational research

## Abstract

Machine learning (ML) has shown its potential to improve patient care over the last decade. In organ transplantation, delayed graft function (DGF) remains a major concern in deceased donor kidney transplantation (DDKT). To this end, we harnessed ML to build personalized prognostic models to predict DGF. Registry data were obtained on adult DDKT recipients for model development (n = 55,044) and validation (n = 6176). Incidence rates of DGF were 25.1% and 26.3% for the development and validation sets, respectively. Twenty-six predictors were identified via recursive feature elimination with random forest. Five widely-used ML algorithms—logistic regression (LR), elastic net, random forest, artificial neural network (ANN), and extreme gradient boosting (XGB) were trained and compared with a baseline LR model fitted with previously identified risk factors. The new ML models, particularly ANN with the area under the receiver operating characteristic curve (ROC-AUC) of 0.732 and XGB with ROC-AUC of 0.735, exhibited superior performance to the baseline model (ROC-AUC = 0.705). This study demonstrates the use of ML as a viable strategy to enable personalized risk quantification for medical applications. If successfully implemented, our models may aid in both risk quantification for DGF prevention clinical trials and personalized clinical decision making.

## Introduction

Delayed graft function (DGF) is an early manifestation of renal allograft injury and is a relatively common complication seen after deceased donor kidney transplantation (DDKT)^1^. While several different dialysis-based and serum creatinine-based definitions for DGF exist today^2^, DGF is often defined as a need for dialysis in the first week following transplantation^3^. In the United States, the incidence rate of DGF reached 21.3% among DDKT patients in 2008, and has seen a moderate increase over the last decade. This is due at least in part to the growing use of expanded criteria donors (ECD) driven by the organ donor shortage burdening the field of organ transplantation^1^. By definition, ECD are kidney donors who are either: (1) age ≥ 60 years; or, (2) age 50 to 59 years with two of the following three criteria: hypertension, terminal serum creatinine > 1.5 mg/dl, or death from cerebrovascular accident^4^. DGF results primarily from ischemia and reperfusion (IR) injury, which is accompanied by acute tubular necrosis in addition to activation of the innate immune system via Toll-like-receptors, inflammasomes, and the complement system. Likewise, adaptive immunity, in which CD4+ and CD8+ T cells are recruited at the site of tissue injury, plays a role in IR injury-induced DGF, exacerbating the progression of IR injury via antigen-specific and antigen-independent pathways^5^. Major risk factors for DGF that have been reported to date include, but are not limited to: increased cold ischemia time, donation after cardiac death (DCD), greater number of human leukocyte antigen (HLA) mismatches, greater recipient body mass index (BMI), longer duration of pre-transplant dialysis, older donor age, and increased donor weight^6,7^. DGF is particularly concerning for both clinicians and DDKT patients as it is associated with as much as a 40% decrease in long-term graft survival^1^, 53% increase in patient death^8^, and 38% increase in the risk of acute rejection^9^, as well as higher economic costs due to prolonged hospital stays^10^.

The deleterious consequences coupled with the moderately high incidence of DGF in DDKT patients necessitate an effort to attenuate the risk and impact of DGF. To this end, several prognostic models have been developed using features available prior to transplant that enable early identification of patients at higher risk of DGF^6,11–13^. Using conventional statistical approaches, these models were constructed by relying primarily on a priori risk factors and multivariate regression techniques. Another attractive modeling approach involves the use of machine learning (ML) in which algorithms learn patterns from data without being explicitly programmed with pre-specified rules. ML, or more broadly, artificial intelligence (AI), has been an active field of research within the field of medicine over the last decade, although it has been around for more than 50 years^14–16^. In transplant settings, the predictive potentials of artificial neural networks (ANN) and tree-based methods such as random forest (RF) have been studied and demonstrated promising results^17–21^. Compared to other industries, healthcare has been tasked with a unique set of challenges in adopting complex ML algorithms due to the need for additional safety and regulatory requirements imposed by the U.S Food and Drug Administration^22–24^. Therefore, more studies are needed in the clinical arena to validate the use of ML as a practical approach for clinical predictive modeling. In this study, we constructed personalized prognostic models with ML techniques to predict DGF in DDKT patients using a large registry database and performed comprehensive validation of the models with a series of statistical techniques. The ML-based prognostic models outperformed a baseline logistic regression model fitted with previously identified risk factors. Successful implementation of our models may potentially assist with (1) development of DGF prevention clinical trials via accurate risk quantification of study subjects; and (2) personalized clinical decision making for DDKT patients.

## Results

### Predictive modeling process

**Figure 1:**
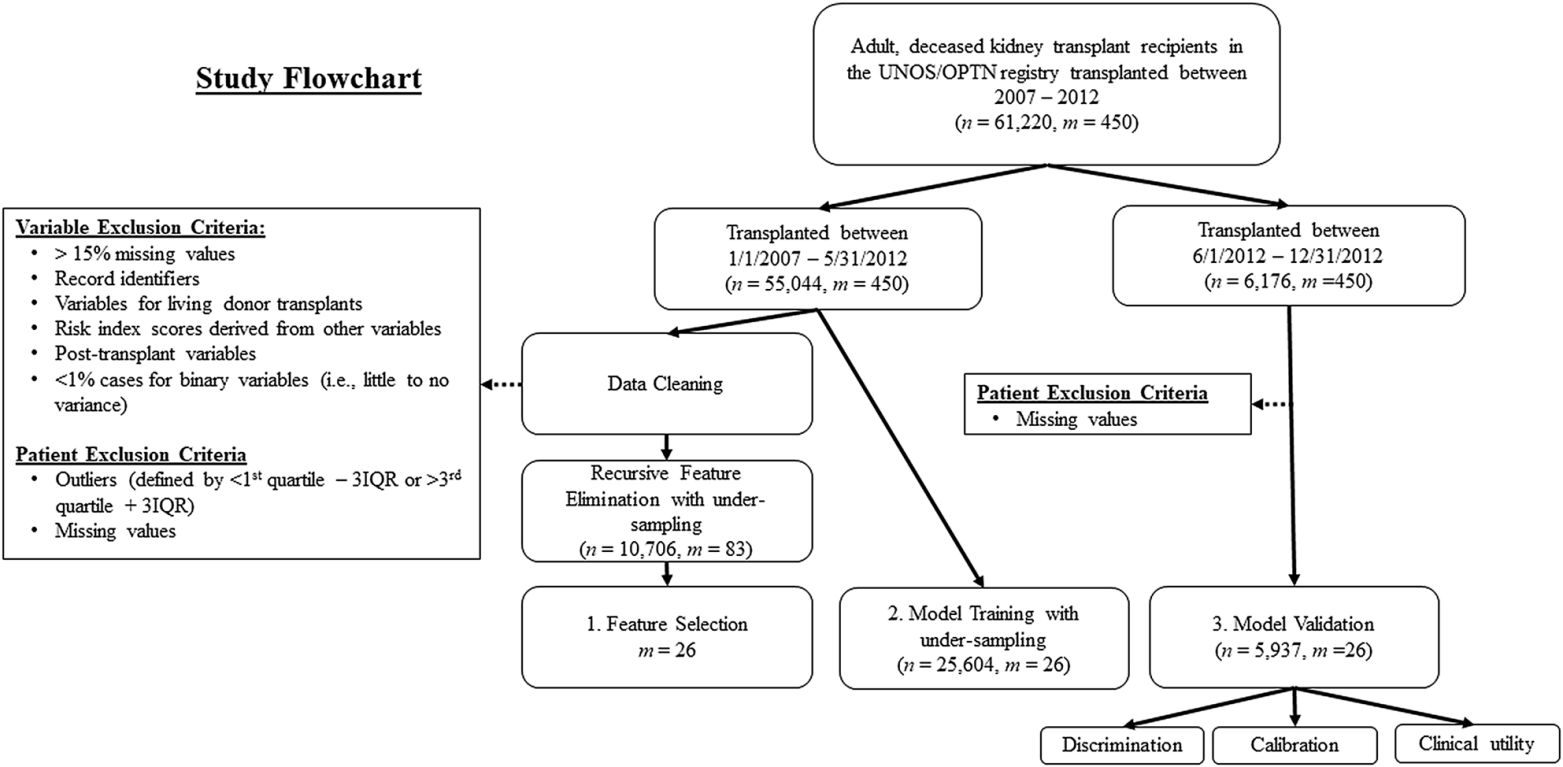
Study design. Data were obtained from the United Network for Organ Sharing/Organ Procurement and Transplantation Network (UNOS/OPTN) on adult deceased donor kidney transplant recipients transplanted between January 1, 2007, and May 31, 2012, for a development set and between June 1, 2012, and December 31, 2012, for a validation set. First, recursive feature elimination with random forest was applied to the data pre-processed as shown. The cleaned dataset was under-sampled to adjust the class distribution, resulting in a dataset of n = 25,604. Five widely-used machine learning algorithms were then trained on the data using tenfold cross-validation. Finally, each model was assessed in the validation cohort for discrimination, calibration, and clinical utility. The letters *n* and *m* represent the numbers of records and features respectively. *IQR* interquartile range.

To develop the ML models, we followed steps as described in Fig. 1. First, data were split into training (development) and validation sets on the transplant date. The training set was then used for recursive feature elimination with random forest (RFE-RF) to calculate the variable importance score (VIS) for each feature and determine an optimal set of predictors using the area under receiver operating characteristic curve (ROC-AUC) as the performance metric. Five ML algorithms were trained with the selected predictors on the training set for which hyper-parameter tuning was done via randomized search with tenfold cross-validation. Finally, the trained models were assessed for overall predictive performance, discrimination, calibration, and clinical utility on the validation set.

### Patient population: development and validation cohorts

The development set included a total of 55,044 patients and the validation set included 6176 patients (Table 1). The two cohorts had comparable characteristics. There were 25.1% and 26.3% DGF, 13.4% and 15.3% DCD donors, and 17.3% and 14.8% ECD kidneys in the development and validation sets, respectively. Overall, the majority of the patients had pre-transplant dialysis (88.5% and 89.2%), were male (60.7% and 60.5%), and were white (45.6% and 44.1%). All of the candidate features selected for feature selection had < 5% missing values with cold ischemia time having the highest % missing values of 2.9%. Prior to model training, the development set was under-sampled to equalize the proportions of patients with and without DGF resulting in 50.0% incidence rate of DGF in the dataset. The under-sampled dataset remained similar with the validation cohort in the rest of the characteristics (Supplementary Table S1).

**Table 1:** Patient demographics: development and validation sets.

	Development set	Validation set	Missing (%)
Date of transplant	01/01/2007–05/31/2012	06/01/2012–12/31/2012	
n	55,044	6176	
**Recipient characteristics**			
Age, mean (SD)	52.76 (13.02)	53.25 (13.19)	0
Male, n (%)	33,411 (60.7)	3735 (60.5)	0
Ethnicity, n (%)			0
White	25,081 (45.6)	2724 (44.1)	
Asian	3300 (6.0)	420 (6.8)	
Black	17,669 (32.1)	1963 (31.8)	
Hispanic	7978 (14.5)	970 (15.7)	
Other	1016 (1.8)	97 (1.6)	
BMI, mean (SD)	28.06 (5.47)	28.44 (5.49)	0.2
Primary diagnosis, n (%)			0.5
Diabetes	14,632 (26.7)	1694 (27.5)	
Hypertension	13,424 (24.5)	1453 (23.6)	
Other	26,728 (48.8)	3007 (48.9)	
Pretransplant dialysis, n (%)	48,444 (88.5)	5500 (89.2)	0.5
Serum creatinine, mean (SD)	7.97 (3.50)	7.97 (3.62)	1
Initial waitlist status other than active, n (%)	9647 (17.5)	1400 (22.7)	0
Diabetes, n (%)			1
No	35,141 (64.5)	3961 (64.4)	
Type I	2680 (4.9)	220 (3.6)	
Type II	14,276 (26.2)	1830 (29.8)	
Type other	223 (0.4)	34 (0.6)	
T ype unknown	2143 (3.9)	103 (1.7)	
Days on waiting list, mean (SD)	858.52 (725.47)	962.87 (774.94)	0.1
**Donor characteristics**			
Age, mean (SD)	38.77 (16.45)	38.18 (16.13)	0
DCD donor, n (%)	7352 (13.4)	944 (15.3)	0
BUN, mean (SD)	16.20 (10.76)	17.27 (13.19)	0.1
Terminal serum creatinine, mean (SD)	1.14 (0.91)	1.13 (0.92)	0
BMI, mean (SD)	27.24 (6.66)	27.56 (6.85)	0.1
History of hypertension, n (%)	15,414 (28.2)	1747 (28.5)	0.6
Cause of death, n (%)			0
Anoxia	13,075 (23.8)	1816 (29.4)	
Cerebrovascular/stroke	19,614 (35.6)	1939 (31.4)	
Head trauma	20,579 (37.4)	2242 (36.3)	
Other	1776 (3.2)	177 (2.9)	
ECD donor, n (%)	9515 (17.3)	911 (14.8)	0
Mechanism of death, n (%)			0
Cardiovascular	6462 (11.7)	838 (13.6)	
Intracranial hemorrhage/stroke	20,334 (36.9)	2003 (32.4)	
Gunshot wound	5596 (10.2)	622 (10.1)	
Blunt injury	13,901 (25.3)	1562 (25.3)	
Other	8751 (15.9)	1149 (18.6)	
History of diabetes, n (%)			0.5
No	50,759 (92.7)	5663 (92.2)	
Type I	2045 (3.7)	226 (3.7)	
Type II	757 (1.4)	96 (1.6)	
Type other	765 (1.4)	107 (1.7)	
Type unknown	455 (0.8)	49 (0.8)	
Arginine vasopressin, n (%)	30,988 (56.4)	3564 (57.8)	0.1
Steroids, n (%)	38,715 (70.5)	4219 (68.3)	0.2
SGPT, mean (SD)	107.35 (346.29)	107.73 (293.77)	0.5
**Transplant characteristics**			
Delayed graft function, n (%)	13,792 (25.1)	1624 (26.3)	0
Allocation type, n (%)			0
Local	41,344 (75.1)	4837 (78.3)	
Regional	4833 (8.8)	550 (8.9)	
National	8867 (16.1)	787 (12.7)	
Cold ischemia time, mean (SD)	17.73 (9.65)	17.14 (8.64)	2.9
Right kidney biopsy at recovery, n (%)	24,659 (44.8)	2929 (47.5)	0.1
Left kidney biopsy at recovery, n (%)	24,370 (44.3)	2907 (47.1)	0
Kidney pump, n (%)	22,378 (40.7)	2801 (45.4)	0

*SD* standard deviation, *BMI* Body Mass Index, *DCD* donation after cardiac death, *BUN* blood urea nitrogen, *ECD* expanded-criteria donation, *SGPT* serum glutamic pyruvic transaminase.

### Identification of predictors

**Figure 2:**
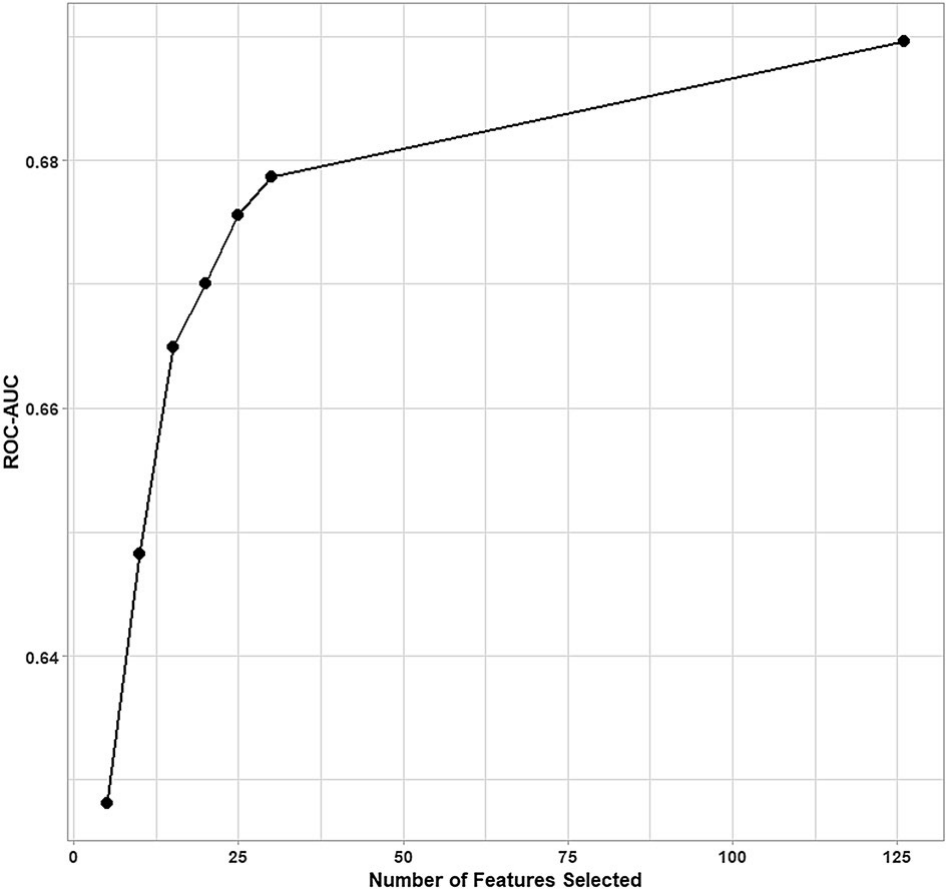
Area under the receiver operating characteristic curve (ROC-AUC) for the tested sets of features. Sets of 5, 10, 15, 20, 25, and 30 features were assessed with ROC-AUC as the performance metric. ROC-AUC for all features (126 features) served as a benchmark. The algorithm reached the highest ROC-AUC when 30 features were used.

After manual screening of the candidate features based on pre-specified exclusion criteria (Fig. 1), RFE-RF was performed on the cleaned dataset, which was under-sampled to adjust for the unequal distribution of those with and without DGF. Categorical features with more than two levels were one-hot-encoded where each level is represented as a dummy variable coded as either one (positive) or zero (negative). This resulted in a total of 126 features. Among the sets of features tested (5, 10, 15, 20, 25, 30), the algorithm yielded the largest ROC-AUC of 0.6786 ± 0.0081 when 30 features were included (Fig. 2). There seemed to be a proportional increase in ROC-AUC with the number of features used up to the maximum number of 126. To ensure model parsimony and facilitate clinical use of the models^25,26^, numbers of features higher than 30 were not considered. The selected 30 features represented the original set of 26 features. Of the 26 predictors, 13 were donor-related, eight were recipient-related, and five were transplant-related (Table 2). The strongest predictor was recipient pretransplant dialysis (VIS = 22.1; Rank = 1). Upon comparison, some of the predictors were found in the baseline predictors^6^ such as recipient pre-transplant dialysis, recipient BMI, recipient black race, recipient diabetes, male recipient, donor age, DCD donor, cold ischemia time, donor terminal serum creatinine, donor history of hypertension, and donor cause of death. In contrast, some features were newly identified as strong predictors of DGF (ranked within top 10), which included recipient serum creatinine (VIS = 9.51; Rank = 3), donor blood urea nitrogen (BUN) (VIS = 8.58; Rank = 5), and right (VIS = 6.87; Rank = 6) and left (VIS = 6.45; Rank = 8) kidney biopsies done at recovery.

**Table 2:** Top 30 predictors selected via recursive feature elimination with random forest algorithm.

Selected predictors	VIS	Rank
Recipient—pretransplant dialysis	22.11	1
Donor—age	9.73	2
Recipient—serum creatinine	9.51	3
Donor—DCD donor	8.95	4
Donor—BUN	8.58	5
Transplant—right kidney biopsy at recovery	6.87	6
Transplant—cold ischemia time	6.66	7
Transplant—left kidney biopsy at recovery	6.45	8
Recipient—BMI	6.12	9
Donor—terminal serum creatinine	5.67	10
Recipient—days on waiting list	5.14	11
Donor—BMI	4.47	12
Recipient—White race	4.27	13
Donor—history of hypertension	3.88	14
Recipient—Black race	3.85	15
Donor—cause of death (trauma)	2.49	16
Recipient—initial waitlist status other than active	2.42	17
Recipient—male	2.30	18
Transplant—kidney pump	2.21	19
Recipient—diabetes (type II)	2.04	20
Recipient—diabetes (type unknown)	1.96	21
Transplant—allocation type (national)	1.82	22
Donor—ECD donor	1.71	23
Donor—mechanism of death (intracranial hemorrhage/stroke)	1.69	24
Donor—history of diabetes (type I)	1.69	25
Donor—arginine vasopressin	1.68	26
Donor—steroids	1.68	27
Donor—SGPT	1.61	28
Donor—cause of death (cardiovascular/stroke)	1.53	29
Donor—mechanism of death (blunt injury)	1.51	30

*BMI* Body Mass Index, *BUN* blood urea nitrogen, *DCD* donation after cardiac death, *ECD* expanded-criteria donation, *SGPT* serum glutamic pyruvic transaminase, *VIS* variable importance score.

### Model development

Next, the development dataset with the selected features was used to train five ML algorithms—logistic regression (LR), elastic net (EN), RF, extreme gradient boosting (XGB), and ANN. To compare performance, a baseline model was developed based on the model published by Irish et al. in 2010. Randomized search with tenfold cross-validation was performed for hyper-parameter tuning. Table 3 shows the mean cross-validated ROC-AUC for each trained model with a set of parameters that resulted in the highest AUC. Baseline (BL) and LR did not have any parameters to optimize. When compared to the BL model (ROC-AUC = 0.703 ± 0.011), all of the trained models scored higher ROC-AUC. The highest AUC was achieved by XGB (ROC-AUC = 0.742 ± 0.009), followed by ANN (ROC-AUC = 0.737 ± 0.007). Of note, the LR model fitted with the new predictors (ROC-AUC = 0.728 ± 0.012) outperformed the baseline LR model (ROC-AUC = 0.703 ± 0.011), suggesting that the selected features are indeed predictive of DGF.

**Table 3:** Hyperparameter tuning via randomized search with tenfold cross-validation.

Model	ROC-AUC ± SD	Number of searches done	Best parameters
BL	0.703 ± 0.011	NA	None
LR	0.728 ± 0.012	NA	None
RF	0.735 ± 0.009	30	mtry = 3
EN	0.728 ± 0.012	100	alpha = 0.883, lambda = 0.00142
XGB	0.742 ± 0.009	100	nrounds = 668, max_depth = 6, eta = 0.0347, gamma = 5.703, subsample = 0.569, colsample_bytree = 0.699, rate_drop = 0.350, skip_drop = 0.805, min_child_weight = 7
ANN	0.737 ± 0.007	100	size = 20, decay = 8.795, number of layer = 1, entropy = TRUE, abstol = 1.0e^−4^, reltol = 1.0e^−8^, maxit = 1.0e^6^

*ROC-AUC* area under the receiver operating characteristic curve, *SD* standard deviation, *BL* baseline, *LR* logistic regression, *EN* elastic net, *RF* random forest, *XGB* extreme gradient boosting, *ANN* artificial neural network.

### Model validation

**Figure 3:**
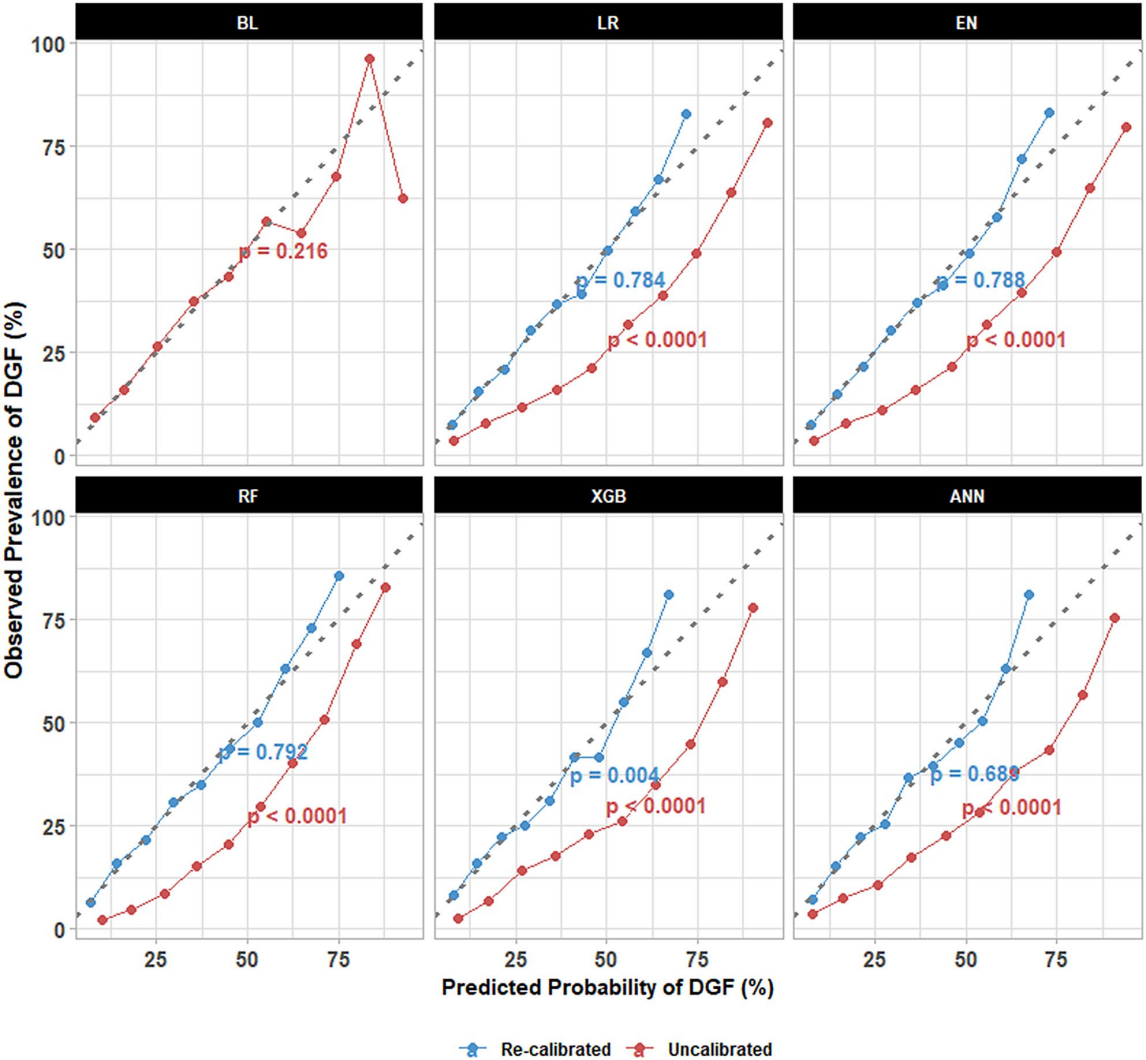
Calibration plots of the uncalibrated (red) versus recalibrated (blue) models. The plots show the observed prevalence of delayed graft function (DGF) versus predicted probability of DGF per decile. The Hosmer–Lemeshow test was performed to test for calibration errors. Only the baseline model had good calibration without recalibration (p > 0.05). When recalibrated with Platt scaling, all of the models showed improvement. *BL* baseline, *LR* logistic regression, *EN* elastic net, *RF* random forest, *XGB* extreme gradient boosting, *ANN* artificial neural network.

**Figure 4:**
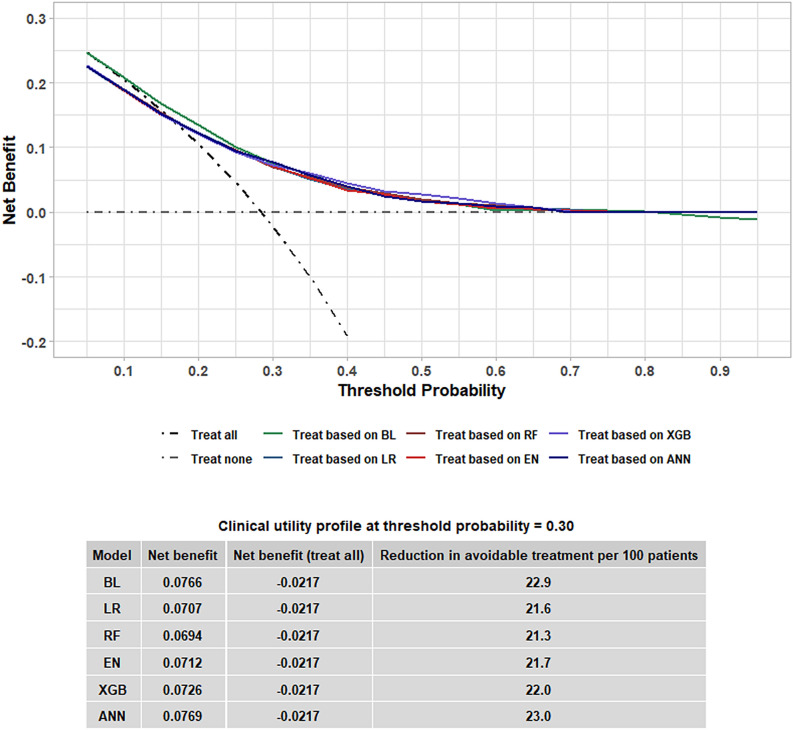
Decision curves of the recalibrated models. Net benefits for threshold probabilities of 0 through 1 with a 0.05 increment are shown for the baseline and recalibrated models. The table shows an example case where a threshold probability of 0.30 is chosen. Reduction in avoidable treatment using each treatment strategy is based on comparison with the “treat all” strategy. *LR* logistic regression, *EN* elastic net, *RF* random forest, *XGB* extreme gradient boosting, *ANN* artificial neural network.

An additional series of analyses was performed for model validation using the validation cohort. The developed models were first tested for calibration. Figure 3 shows the calibration plots of the models before and after recalibration via Platt scaling. Most of the models overestimated the probability of DGF, but after recalibration, all of the calibration curves were more closely aligned with the 45 degree line; the recalibration technique improved the model calibration. The baseline model had good calibration (p = 0.216) and did not require any recalibration although the line showed a degree of inconsistency towards the higher end of the line (higher probability of DGF). The deviations observed for the upper deciles may be due to the smaller sample size. Additionally, the mean predicted probability of DGF from the recalibrated models closely matched with the observed prevalence of DGF in different risk groups (Supplementary Table S2). While all of the recalibrated models performed better than BL (Brier score = 0.182), XGB achieved the highest overall performance with a Brier score of 0.167 and ∆Brier score of − 0.015 (Table 4, Supplementary Table S3). Next, model discrimination was evaluated with ROC-AUC, precision-recall AUC (PR-AUC), and integrated discrimination improvement (IDI). The results are summarized in Table 4, Supplementary Figs. S1, S2 and Table S3. XGB had the highest ROC-AUC of 0.735 and PR-AUC of 0.519 followed by ANN with ROC-AUC of 0.732 and PR-AUC of 0.498. Differences in ROC-AUC compared with BL as denoted by ∆ROC-AUC were + 0.031 (p = 0.005) and + 0.027 (p = 0.012), and ∆PR-AUC were + 0.033 and + 0.012 for XGB and ANN, respectively. Likewise, XGB (IDI = 0.025; discrimination slope = 0.142) and ANN (IDI = 0.018; discrimination slope = 0.135) had the largest discrimination slopes and IDI, whereas the baseline discrimination slope was 0.117. Finally, clinical utility of the recalibrated models was evaluated with decision curve analysis. This model validation technique quantifies the net benefit associated with some hypothetical treatment given for a range of threshold probabilities used to determine which patients need to receive the treatment (Fig. 4). For threshold probabilities between 0.20 and 0.60, all of the models showed a degree of net benefit that is higher than that of a strategy where all DDKT patients are treated for DGF. As an example, at a threshold value of 0.30, the ANN model had the highest net benefit of 0.0769 and “treat all” has a net benefit of − 0.0217, which translates to a reduction in avoidable treatments by 23.0 per 100 DDKT patients. Interestingly, at this threshold probability, the net benefit for BL surpassed those of most of the newly developed models despite having poorer discrimination. The exact cut-off value to be used will vary depending on the degree of harm associated with unnecessary treatment of DGF (i.e., false positives). Lower threshold values are recommended for relatively less harmful treatments and vice versa.

**Table 4:** Differences in performance compared with the baseline model.

Model	∆Brier Score	IDI	∆PR-AUC	∆ROC-AUC	DeLong test
LR	− 0.012	+ 0.011	+ 0.006	+ 0.021	p = 0.056
RF	− 0.013	+ 0.017	+ 0.011	+ 0.026	p = 0.018
EN	− 0.012	+ 0.011	+ 0.005	+ 0.021	p = 0.056
XGB	− 0.015	+ 0.025	+ 0.033	+ 0.031	p = 0.005
ANN	− 0.013	+ 0.018	+ 0.012	+ 0.027	p = 0.012

*IDI* integrated discrimination improvement, *PR-AUC* area under the precision-recall curve, *ROC-AUC* area under the receiver operating characteristic curve, *SD* standard deviation, *BL* baseline, *LR* logistic regression, *EN* elastic net, *RF* random forest, *XGB* extreme gradient boosting, *ANN* artificial neural network.

## Discussion

With the growing use of ECD fueled by a donor organ shortage, DGF has become a more significant concern among the transplant community^2^. To this end, several groups have developed scoring systems that enable clinicians to identify patients at higher risk of developing DGF at an early stage^6,11–13^. While multivariate LR and Cox regression are considered standard methods to develop a scoring system for risk quantification, ML is another predictive modeling approach. We would like to clarify that throughout the manuscript, LR is referred to as a ML algorithm, however, the appropriate classification of LR is context-dependent and depends upon whether it is used for prediction (ML) or inferential statistics to evaluate associations between the independent variable(s) and dependent variable (non-ML). ML has recently seen a surge of interest in various industries, including the healthcare industry, owning to advances in Big Data technology and computing power^15^. In a recent study, the authors compared the predictive ability of LR with that of several ML algorithms for DGF and showed that support vector machine (SVM) with a linear-basis function kernel had superior performance compared to the rest of the algorithms. However, the study used data collected from a single center (n = 497) and therefore, there is a possibility of overfitting by the model, rendering its generalizability questionable^19^. In the current study, we developed ML models using the United Network for Organ Sharing/Organ Procurement and Transplantation Network (UNOS/OPTN) registry, a national-scale database for organ transplantation (n = 61,220) and performed comprehensive validation of the models. To our knowledge, this is the first study to develop multiple ML models for DGF prediction using a dataset of this size and features selected via RFE-RF. Moreover, we included patient subpopulations that were excluded in the previous study by Irish et al. and therefore, our models may be applicable to a larger patient population. We did not include SVM in the final model development process as our preliminary results indicated that SVM only performed marginally better on a similar, but smaller dataset^27^. Furthermore, we experienced extremely long model training time due to the size of our dataset and computational complexity of SVM, which is known to be ≈ O(n^2^), where n is the sample size^28^.

After training the ML algorithms, we assessed each model for three performance measures: discrimination, calibration, and clinical utility, with the latter two being less common but essential for clinical model validation^29^. All of the algorithms trained with the new predictors performed better or equally well in these aspects compared to the BL model, especially ANN and XGB. It is noteworthy that better model discrimination did not always indicate superior clinical utility as observed for XGB. This may be explained by the fact that the decision curve analysis as proposed by Vickers et al.^30^ does not consider the net benefit of those who are not treated based on the models. Consistent with our findings, ANN has previously been demonstrated to be superior to LR in predicting transplant outcomes including DGF using single-center data^21,31,32^. ANN with one or more hidden layers is different from LR in that the hidden layers in ANN perform data abstraction and send the output to a final classification layer. This makes the algorithm capable of “learning” non-linear relationships between the independent and dependent variables^33^. On the other hand, LR traditionally is an algorithm of choice for linear classification problems^34^. This is one plausible explanation as to why our ANN model surpassed the baseline and new LR models. Likewise, XGB is an ensemble learning method, which assembles decision trees as its building blocks to build a strong learner that is able to learn nonlinear relationships between predictors and outcome^35^. XGB has recently been shown to have superior predictive performance to other ML algorithms in various contexts^36–39^.

Another important factor is the feature selection step. Previously, selection of risk factors was done generally by assessing preselected features in generalized linear models such as multivariate LR and generalized additive models, which is another statistical method capable of modeling non-linearity^40,41^. Here, we utilized RFE-RF instead, which allows for extraction of relevant features from a large pool of features in order to optimize the final predictive performance. Further, RF have a non-linear decision boundary and are considered to be a non-parametric method that is relatively robust to outliers making RFE-RF a versatile technique for feature selection^42,43^. Therefore, the success of our ML models is presumably attributed to the minimal yet sufficient manual elimination of features from the candidate pool and the subsequent feature selection by RFE-RF, which minimizes our dependence on a priori knowledge.

The feature selection process with RFE-RF revealed a total of 26 features as predictors of DGF. The most potent predictor was recipient pretransplant dialysis for which studies have shown a significant association with elevated risk of DGF^6,7,44^. Interestingly, there are some factors that are not found in the baseline predictors, but were identified as strong predictors of DGF and ranked within top 10 based on the VIS. These new predictors include recipient serum creatinine, donor BUN, and kidney biopsies done at recovery. Serum creatinine is widely used as an indicator of renal function in clinical practice and serves as a biomarker to monitor the allograft status^45^. While elevated levels of serum creatinine are often associated with compromised renal function, patients with a higher pre-transplant serum creatinine level, which is a surrogate of larger muscle mass, tend to have better post-transplant graft and patient survival^46^. Similar to serum creatinine, BUN is commonly used clinically as a measure of renal function, and higher BUN concentrations are indicative of kidney dysfunction^47^. Procurement biopsies are performed in about 50% of deceased donor kidneys in the Unites States for DDKT to assess the quality of donor organs, and needle biopsies are thought to increase the risk of bleeding post-transplantation^48,49^. Irish et al. excluded machine-perfused kidneys from their study cohort as they may complicate the analysis of risk factors. However, we found that the use of kidney pump is predictive of DGF (VIS = 2.21, Rank = 19), and prior studies reported a decreased risk of DGF associated with machine-perfused kidneys^50–52^. We did not consider any feature sets larger than 30 features in our study as we realize the concept of model parsimony is one of the critical aspects of building clinically useful models^53^. It is also important to remember that correlation does not always indicate causation, and the feature selection method only suggests that these features are predictive of DGF with a “potential” causal relationship with the outcome.

While ML has gained increasing attention in the healthcare industry, there are concomitant bioethical concerns surrounding the use of complex ML algorithms as they tend to have poor interpretability^22,24^. This has led to the preferred use of algorithms with high model transparency such as decision trees and LR. However, more complex models have been shown to predict clinical outcomes with higher accuracy and are capable of handling unstructured data such as images and electric medical records more efficiently^54^. Thus, more research is needed to better ascertain AI’s capability and delineate where AI fits in medicine. AI has the potential to assist in the areas of diagnosis, treatment, and clinical workflow to augment the work of clinicians^54,55^. This synergy between human and ML in clinical settings suggests that the implementation of AI may be key to making high quality patient care more accessible to a larger population. Establishing the right balance of human intervention and AI will likely be of utmost importance to maximize AI’s potential in this field. In addition to the healthcare arena, ML models may become valuable tools for the pharmaceutical industry and clinical researchers in order to increase success rates of clinical trials^56^. Clinical trials for drug development consist of lengthy processes that consume substantial amounts of resources and efforts. Consequently, strategies to reduce trial failures are imperative. ML algorithms, if trained and validated properly, may be part of such strategies to aid in patient stratification, treatment response identification, and/or subgroup identification^57^.

One of the limitations of this study is that we were unable to include warm ischemia time and peak calculated panel reactive antibody (cPRA) in our baseline model, which could be another explanation for its lower predictive score observed in our study. Furthermore, Irish et al. included recipients transplanted in a different time period rendering apple-to-apple comparisons impossible. However, it needs to be emphasized that the primary objective of this study is not to demonstrate one approach is better than the other, but to propose ML as an alternative method to build a clinically useful tool. In fact, external validation studies of existing predictive models for DGF were conducted in Dutch^58^ and Chinese^59^ cohorts separately, and the model developed by Irish and his colleagues outperformed the other models in both studies. Our study would also benefit from external validation in non-UNOS/OPTN data and further analysis with external validation data is forthcoming. Another potential limitation of this study is that we did not perform in-depth analyses of the algorithms and selected predictors when both of the best performing models in our study (ANN and XGB) are considered black box algorithms. Therefore, the future direction is to further ensure that the predictions are sensible and that the models are explainable using techniques such as local interpretable model-agnostic explanations and Shapley additive explanations among others^60^. Furthermore, we will assess how model performance changes with fewer predictors in an attempt to reduce the number of predictors needed and improve model parsimony.

We have demonstrated here that ML is a valid alternative approach for prediction and identification of predictors of DGF, adding an important piece of evidence to support the use of ML to drive medical advancements. Additional effort to improve model interpretability and transparency will be essential to expedite the successful implementation and use of complex yet high-performing ML algorithms for clinical applications. If properly implemented, our prognostic systems may potentially be used to augment the workflows in clinics and drug development for DGF.

## Materials and methods

### Study design

We obtained de-identified data from the UNOS/OPTN standard transplant analysis and research files on adult DDKT recipients transplanted between January 1, 2007, and May 31, 2012. This timeframe was selected to train ML models with large and more recent data than the original study by Irish et al. and to allow 3 years for data entry to ensure completeness of data. This dataset was used for feature selection and model development. The patient cohort consisted of all DDKT patients, including single organ and simultaneous multiple organ transplants, pre-emptive and non-preemptive transplants, and machine-perfused and non-machine perfused kidneys. DGF was defined as a need for dialysis within the first week following transplant. Patients in the UNOS/OPTN database who received a renal transplant between June 1, 2012, and December 31, 2012, were selected as a validation cohort (Fig. 1).

### Selection of predictors

First, 450 pre-transplant features for kidney transplantation available in UNOS/OPTN data were manually screened using the following exclusion criteria: post-transplant features, risk scores, subject identifiers, features with greater than 15% missing data, and living donor transplant variables. This pre-screening step resulted in 83 candidate features (Fig. 1), which increased to 126 after categorical features were one-hot-encoded. In order to address the class imbalance problem with approximately 25% incidence rate of DGF, under-sampling of data was performed to adjust the distribution of patients with and without DGF. After removal of records with missing values and/or outliers, RFE-RF was applied to the processed dataset to reveal the predictors for DGF. In brief, RFE-RF ranks features by VIS, which is calculated based on final predictive accuracy and determines the optimal number of predictors in an arbitrarily pre-defined search space^61^. In this study, we tested 5, 10, 15, 20, 25, 30, and 126 features with tenfold cross-validation and used mean ROC-AUC as the performance metric.

### Model development

As was done for feature selection, under-sampling was performed to combat the class imbalance problem. All continuous variables were standardized where needed. The model training and hyper-parameter tuning were done using randomized search with tenfold cross-validation and mean ROC-AUC as the performance metric. Five commonly used ML algorithms were trained including LR, RF, EN, ANN, and XGB. These algorithms were implemented in R using the following R packages: stats, randomForest, glmnet, nnet, and xgboost, respectively.

LR is one of the most commonly used ML algorithms when the dependent variable is categorical with a binominal distribution^62^. LR is highly interpretable as a unique contribution of each variable can be easily quantified with beta coefficient. In R, as implemented by the glm function, the algorithm estimates the parameters using the Fisher scoring algorithm, also known as iteratively reweighted least squares for maximum likelihood estimation. The loss function (log loss) to be minimized can be expressed as:

$$Log\, loss= -{\sum }_{i=1}^{n}{y}_{i}\text{ln}{p}_{i}+\left(1-{y}_{i}\right)\text{ln}\left(1-{p}_{i}\right)$$

where *n* is the number of observations, *p*_*i*_ is the predicted probability for ith individual, and *y*_*i*_ is ith observed outcome^63^.

EN is a regularization method, which simultaneously applies the ridge penalty (L1) and Last Absolute Shrinkage and Selection Operator penalty (L2) to penalize the parameters and reduce overfitting. Consequently, for regularized LR, the loss function is similar to the one given above, but modified to include both L1 and L2 regularization terms as follows:

$$Regularized \,Log \,loss = Log \,loss + {\alpha \lambda }{\sum }_{j=1}^{p}{\upbeta }_{\text{j}}^{2}+ (1-{\alpha })\uplambda {\sum }_{j=1}^{p}|{\upbeta }_{\text{j}}|$$

where *p* is the number of parameters, α is a L1/L2 weighting factor, and λ is a shrinkage parameter^63,64^.

RF is an ensemble learning algorithm, in which “deep” decisions trees are built in parallel and aggregated at the end to reduce variance, a concept known as bagging. While there exist different forms of RF, we selected the original version of RF as proposed by Breiman et al.^65^, where each decision tree is built using a bootstrapped sample and fed with a randomly selected set of features. The trees were constructed with the decrease in the Gini Impurity index as the splitting rule where the index is defined as:

$$Gini \,index=1- {\sum }_{i =1}^{C}{\left({p}_{i}\right)}^{2}$$

where *c* is the number of classes for the feature being split on and *p* is the proportion of class *i* in the node^66^.

Gradient boosting with decision trees is another ensemble method where the base learners (i.e., “shallow” decision trees) are combined sequentially rather than in parallel to reduce bias to build a strong learner. In the most generic form, the algorithm iteratively fits a base learner to the training dataset and estimates the step length ($\upgamma$) that will be used to update the model (F_m−1_(x)) in accordance with the following formula:

$${\upgamma }_{\text{m}}=argmi{n}_{\upgamma }{\sum }_{i-1}^{n}L({y}_{i}, {F}_{m-1}\left({x}_{i}\right)+ \gamma {h}_{m}\left({x}_{i}\right))$$

$${\text{F}}_{\text{m}}\left(x\right)={F}_{m-1}\left(x\right)+ {\upgamma }_{\text{m}}{h}_{m}(x)$$

where *L* is a loss function, *h*_*m*_is a base learner, *n* is the number of observations, and *m* is the number of iterations. The minimization problem is solved by a steepest descent algorithm^67^. In our current study, XGB was used as it is recognized as one of the most efficient implementations of gradient boosting. Compared with gradient boosting machine, another implementation of gradient boosting, XGB is generally faster, has more regularization options, and adds more randomness to features selected to build the trees^35^.

ANN is an algorithm that mimics the human brain to perform classification/prediction tasks. The ANN topology typically consists of three distinct types of layers: an input layer, one or more hidden layers, and an output layer. The input nodes receive a vector of feature from training data and are interconnected to the hidden layer with a set of weights associated with the connections. This intermediate layer, which sends the processed signal to the output nodes enables the algorithm to learn non-linearity between the input features and output. In our implementation of ANN, the Broyden–Fletcher–Goldfarb–Shanno algorithm was used to solve the optimization problem to minimize the binary cross entropy, which is equivalent to the log loss function introduced earlier. This optimization involves an iterative process where the weights are updated to minimize the cost function until the discrepancies fall below a pre-specified tolerance criterion^33,34^.

For comparisons, a baseline model was constructed by training a logistic regression model with a set of predictors identified by Irish et al. in 2010^6^: most recent cPRA, duration of dialysis, recipient BMI, number of HLA mismatches, cold ischemia time, donor terminal creatinine, donor age, donor weight, black recipient, male recipient, previous transplant, recipient diabetes, recipient pre-transplant transfusion, DCD, donor history of hypertension, and donor cause of death. The original model had peak cPRA and warm ischemia time, but the former was replaced with most recent cPRA and the latter was not included in our baseline model as the data were not available.

### Model validation and evaluation

Model validation was conducted using the validation set. Overall predictive performance and discrimination were evaluated using the Brier score, ROC-AUC, PR-AUC, discrimination slope, and IDI^68^. The Brier score is a measure of both calibration and discrimination and takes the squared differences between binary outcomes (0 or 1) and predicted probabilities (0 to 1) with the value ranging from 0 (a perfect model) to 1 (a non-informative model). ROC-AUC is the area under the ROC curve, which is a plot of the true positive rate versus false positive rate for all possible threshold probabilities. PR-AUC is much like ROC-AUC, but the curve shows the precision (positive predictive value) versus the recall (true positive rate), and is more sensitive to correct prediction of the event (positive) class when the binary outcome variable has a skewed distribution^69^. The discrimination slope has emerged relatively recently as a measure to assess discrimination, and is defined as a difference in the mean predicted probability between event and non-event classes. In addition to differences in ROC-AUC and PR-AUC, change in the discrimination slope, IDI was employed to quantify improvement in performance compared with the baseline model^70,71^. Calibration was assessed with the calibration plot, in which the observed prevalence of DGF was plotted against the mean predicted probability of DGF per decile. Poorly calibrated models were recalibrated via Platt scaling by fitting a new logistic regression model with the unadjusted probability values^29^. Clinical usefulness of the models was assessed via decision curve analysis^30^. To develop the decision curves, the net benefits were plotted against threshold probabilities of zero through one with an increment of 0.05 for three different treatment strategies: all patients are treated, no patients are treated, and only selected patients are treated for DGF using the prognostic systems. The net benefit was calculated as follows:

$$NB=\frac{TP}{n}-\frac{FP}{n}\left(\frac{{p}_{t}}{1-{p}_{t}}\right)$$

where *NB* = net benefit, *TP* = true positive count, *n* = sample size, *FP* = false positive count, and *p*_*t*_ = threshold probability. Reduction in avoidable treatment per 100 patients was then computed by:

$$\left(N{B}_{m}-N{B}_{treat all}\right)\left(\frac{{p}_{t}}{1-{p}_{t}}\right)\times100$$

where *NB*_*m*_ = net benefit of the model and *NB*_*treat* all_ = net benefit of the “treat all” strategy.

### Statistical analysis

The Wilcoxon rank-sum test was used to compare the medians of predicted probabilities between those with and without DGF. The Hosmer–Lemeshow test was performed for evaluation of calibration errors. Where appropriate, continuous variables are expressed with mean and standard deviation, and categorical variables with count and percentages. A significance level of 0.05 was used to determine statistical significance unless otherwise stated.

All statistical analyses and development of ML models were performed using R version 3.5.1: A language and environment for statistical computing. R Foundation for Statistical Computing, Vienna, Austria. (https://www.R-project.org).

## Supplementary information

Supplementary information.

## Data Availability

The authors do not own the data, which were used under license for the current study. All relevant data are available from the UNOS/OPTN. Interested researchers may request access to the Standard Transplant Analysis and Research (STAR) file, which contains de-identified information on all transplants performed since 1987 via the online form (https://optn.transplant.hrsa.gov/data/request-data/).
