# Supplementary material for: Personalized prediction of delayed graft function for recipients of deceased donor kidney transplants with machine learning
Source: Sci Rep. 2020 Oct 27;10:18409. doi: 10.1038/s41598-020-75473-z (PMC7591492; doi:10.1038/s41598-020-75473-z)

# **Title**

Personalized prediction of delayed graft function in recipients of deceased donor kidney transplants with machine learning

# **Authors**

Satoru Kawakita*, Jennifer L. Beaumont, Vadim Jucaud, Matthew J. Everly

# **Affiliation**

Terasaki Research Institute, Los Angeles, California, USA

# **Funding**

This work was supported in part by Health Resources and Services Administration contract 234-2005-37011C. The content is the responsibility of the authors alone and does not necessarily reflect the views or policies of the Department of Health and Human Services, nor does mention of trade names, commercial products, or organizations imply endorsement by the U.S. Government.

# **Corresponding Author**

Satoru Kawakita

Contact information: [skawakita@terasaki.org](mailto:skawakita@terasaki.org)

# **Table S1. Patient demographics: under-sampled development set and validation set.**

|  | **Development** | **Validation** | **Missing (%)** |
| --- | --- | --- | --- |
| Date of Transplant | 01/01/2007-05/31/2012 | 06/01/2012-12/31/2012 |  |
| n | 25,604 | 6,176 |  |
| **Recipient** |  |  |  |
| Age, mean (SD) | 52.97 (12.84) | 53.25 (13.19) | 0 |
| Male, n (%) | 16,139 (63.0) | 3,735 (60.5) | 0 |
| Ethnicity, n (%) |  |  | 0 |
| White | 10,975 (42.9) | 2,724 (44.1) |  |
| Asian | 1,506 ( 5.9) | 420 ( 6.8) |  |
| Black | 8,853 (34.6) | 1,963 (31.8) |  |
| Hispanic | 3,791 (14.8) | 970 (15.7) |  |
| Other | 479 ( 1.9) | 97 ( 1.6) |  |
| BMI, mean (SD) | 28.39 (5.54) | 28.44 (5.49) | 0 |
| Primary diagnosis, n (%) |  |  | 0.4 |
| Diabetes | 7,305 (28.7) | 1,694 (27.5) |  |
| Hypertension | 6,350 (24.9) | 1,453 (23.6) |  |
| Other | 11,837 (46.4) | 3,007 (48.9) |  |
| Pretransplant dialysis, n (%) | 23,345 (91.2) | 5,500 (89.2) | 0 |
| Serum creatinine, mean (SD) | 8.21 (3.47) | 7.97 (3.62) | 0.1 |
| Initial waitlisting status other than active, n (%) | 4,735 (18.5) | 1,400 (22.7) | 0 |
| Diabetes, n (%) |  |  | 0.1 |
| No | 15,980 (62.4) | 3,961 (64.4) |  |
| Type I | 1266 ( 4.9) | 220 ( 3.6) |  |
| Type II | 7,177 (28.0) | 1,830 (29.8) |  |
| Type Other | 97 ( 0.4) | 34 ( 0.6) |  |
| Type Unknown | 1084 ( 4.2) | 103 ( 1.7) |  |
| Days on waiting list, mean (SD) | 888.95 (729.54) | 962.87 (774.94) | 0 |
| **Donor** |  |  |  |
| Age, mean (SD) | 39.79 (16.20) | 38.18 (16.13) | 0 |
| DCD donor, n (%) | 4038 (15.8) | 944 (15.3) | 0 |
| BUN, mean (SD) | 17.06 (11.43) | 17.27 (13.19) | 0 |
| Terminal serum creatinine, mean (SD) | 1.19 (0.99) | 1.13 (0.92) | 0 |
| BMI, mean (SD) | 27.61 (6.82) | 27.56 (6.85) | 0 |
| History of hypertension, n (%) | 7780 (30.4) | 1,747 (28.5) | 0.1 |
| Cause of death, n (%) |  |  | 0 |
| Anoxia | 6,320 (24.7) | 1,816 (29.4) |  |
| Crebrovascular/Stroke | 8,981 (35.1) | 1,939 (31.4) |  |
| Head trauma | 840 ( 3.3) | 2,242 (36.3) |  |
| Other | 9,463 (37.0) | 177 ( 2.9) |  |
| ECD donor, n (%) | 4,794 (18.7) | 911 (14.8) | 0 |
| Mechanism of death, n (%) |  |  | 0 |
| Cardiovascular | 3,228 (12.6) | 838 (13.6) |  |
| Gunshot wound | 2,401 ( 9.4) | 622 (10.1) |  |
| Blunt injury | 6,117 (23.9) | 1,562 (25.3) |  |
| Other | 4,109 (16.0) | 1,149 (18.6) |  |
| Intracranial hemorrhage/Stroke | 9,749 (38.1) | 2,003 (32.4) |  |
| History of diabetes, n (%) |  |  | 0.1 |
| No | 23,625 (92.3) | 5,663 (92.2) |  |
| Type I | 1,023 ( 4.0) | 226 ( 3.7) |  |
| Type II | 389 ( 1.5) | 96 ( 1.6) |  |
| Type Other | 350 ( 1.4) | 107 ( 1.7) |  |
| Type Unknown | 217 ( 0.8) | 49 ( 0.8) |  |
| Arginine vasopressin, n (%) | 13,943 (54.5) | 3,564 (57.8) | 0 |
| Steroids, n (%) | 17,707 (69.2) | 4,219 (68.3) | 0 |
| SGPT, mean (SD) | 113.72 (377.81) | 107.73 (293.77) | 0.1 |
| **Transplant** |  |  |  |
| Delayed graft function, n (%) | 12,802 (50.0) | 1,624 (26.3) | 0 |
| Allocation type, n (%) |  |  | 0 |
| Local | 18,860 (73.7) | 4,837 (78.3) |  |
| Regional | 2,396 ( 9.4) | 550 ( 8.9) |  |
| National | 4,348 (17.0) | 787 (12.7) |  |
| Cold ischemia time, mean (SD) | 18.19 (9.54) | 17.14 (8.64) | 0.2 |
| Right kidney biopsy at recovery, n (%) | 12,461 (48.7) | 2,929 (47.5) | 0 |
| Left kidney biopsy at recovery, n (%) | 12,301 (48.0) | 2,907 (47.1) | 0 |
| Kidney pump, n (%) | 10,441 (40.8) | 2,801 (45.4) | 0 |

BMI, body mass index; BUN, blood urea nitrogen; DCD, donation after cardiac death; ECD, expanded-criteria donation; SD, standard deviation; SGPT, serum glutamic pyruvic transaminase.

# **Table S2. Mean predicted probability of delayed graft function (DGF) versus observed prevalence of DGF in different risk groups of the validation cohort.**

|  | **Expanded criteria donors (n=877)** | | **Donation after cardiac death (n=916)** | | **Recipient pretransplant dialysis (n=5,295)** | |
| --- | --- | --- | --- | --- | --- | --- |
| **Model** | **% Observed** | **% Predicted ± SD** | **% Observed** | **% Predicted ± SD** | **% Observed** | **% Predicted ± SD** |
| BL* | 31.6 | 32.8±12.9 | 43.1 | 44.6±15.5 | 28.5 | 28.2±16.0 |
| LR | 30.3 | 30.9±17.5 | 41.2 | 41.3±17.2 | 28.6 | 28.6±18.0 |
| RF | 30.3 | 30.9±14.2 | 41.2 | 40.9±14.8 | 28.6 | 28.6±15.3 |
| EN | 30.3 | 30.8±16.9 | 41.2 | 41.5±16.7 | 28.6 | 28.6±17.5 |
| XGB | 30.3 | 30.3±18.8 | 41.2 | 40.6±20.1 | 28.6 | 28.6±20.0 |
| ANN | 30.3 | 30.4±18.5 | 41.2 | 40.9±19.0 | 28.6 | 28.6±19.5 |

*BL had a different set of predictors with different amounts of missing data. As such, sample sizes were similar but different for expanded criteria donors (n=728), donation after cardiac death (n=761), and recipient pretransplant dialysis (n=4,866). SD, standard deviation; BL, baseline; LR, logistic regression; EN, elastic net; RF, random forest; XGB, extreme gradient boosting; ANN, artificial neural network.

# **Figure S1. The area under the receiver operating characteristic curve (ROC-AUC) and precision-recall AUC (PR-AUC) of the models in the validation set.** Compared with the baseline model, the machine learning models, particularly XGB and ANN, demonstrated higher ROC-AUC and PR-AUC. BL, baseline; LR, logistic regression; EN, elastic net; RF, random forest; XGB, extreme gradient boosting; ANN, artificial neural network.


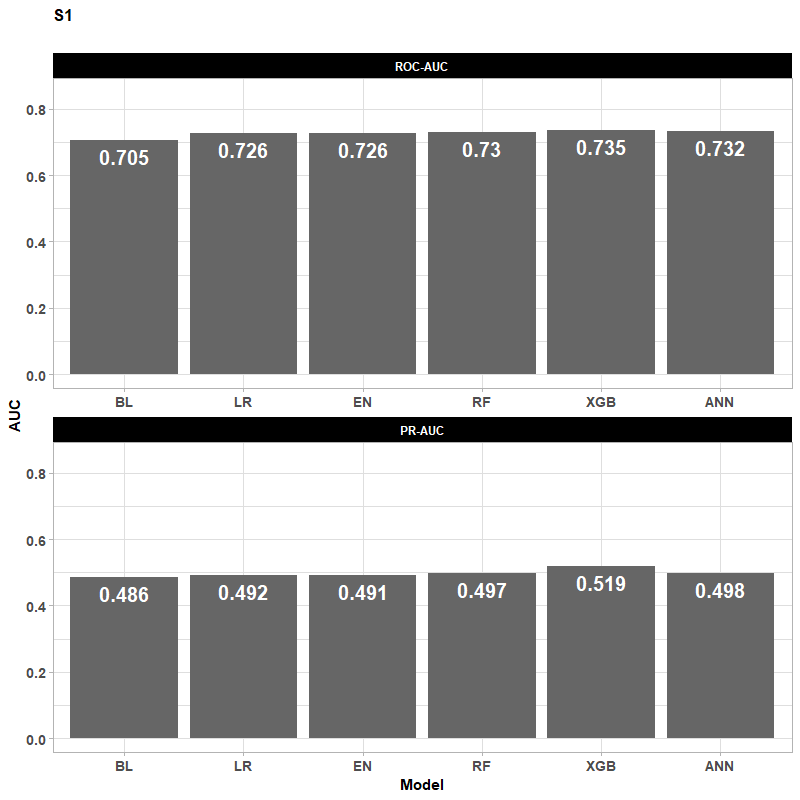


# **Table S3. Brier score, mean predicted probability of delayed graft function (DGF) and discrimination slope in the validation set.**

| **Model** | **Mean Predicted Probability in DGF-** | **Mean Predicted Probability in DGF+** | **Discrimination Slope** | **Brier Score** |
| --- | --- | --- | --- | --- |
| BL | 0.249 | 0.366 | 0.117 | 0.182 |
| LR | 0.230 | 0.359 | 0.128 | 0.169 |
| RF | 0.229 | 0.363 | 0.134 | 0.168 |
| EN | 0.230 | 0.359 | 0.128 | 0.169 |
| XGB | 0.227 | 0.369 | 0.142 | 0.167 |
| ANN | 0.229 | 0.363 | 0.135 | 0.168 |

BL, baseline; LR, logistic regression; EN, elastic net; RF, random forest; XGB, extreme gradient boosting; ANN, artificial neural network.

# **Figure S2. Boxplots showing the distribution of predicted probability of delayed graft function (DGF) in the validation cohort.** The Wilcoxon rank-sum test was performed to compare the medians between DGF positive and DGF negative strata. The median predicted probability was higher in DGF+ patients compared to DGF- patients for all models including the baseline. The dot within each bar represents the mean, which was used to compute the discrimination slope and integrated discrimination improvement. BL, baseline; LR, logistic regression; EN, elastic net; RF, random forest; XGB, extreme gradient boosting; ANN, artificial neural network.


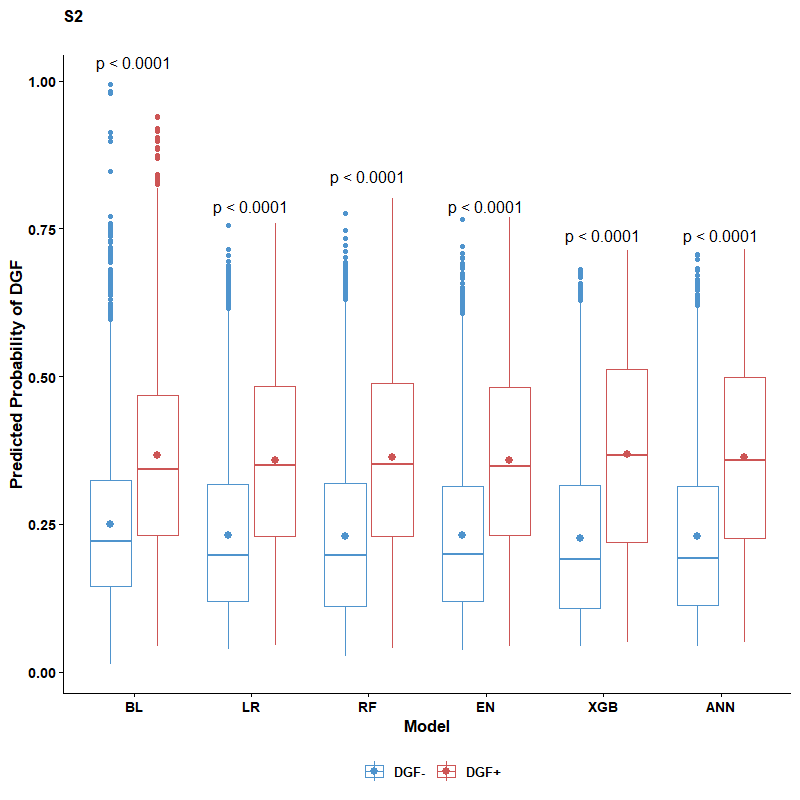

Supplement: Supplementary file 1 — Supplementary information. [file 41598_2020_75473_MOESM1_ESM.docx]
